# Beyond susceptibility testing: traditional Chinese medicine-associated compounds as candidate antibiotic adjuncts in chronic-wound biofilms

**DOI:** 10.3389/fcimb.2026.1991374

**Published:** 2026-10-02

**Authors:** Nan Lu, Ru-ping Zhao, Jun-Jie He, Yi-Ping Zhou, Jin-Sheng Zhang

**Affiliations:** 1 The Affiliated Traditional Chinese Medicine Hospital, Guangzhou Medical University, Guangzhou, China; 2 Guangzhou University of Chinese Medicine, Guangzhou, China

**Keywords:** antibiotic adjunct, antibiotic tolerance, biofilm, chronic wound infection, diabetic foot infection, persister-like cells, small-colony variants, traditional Chinese medicine

## Abstract

Chronic-wound infections may respond poorly even when routine testing shows that the infecting organism remains susceptible. In tissue, bacteria occupy aggregates shaped by oxygen and nutrient gradients, host material, diabetes-associated chemistry and uneven antibiotic exposure. These conditions can slow killing and favour small-colony variants or intracellular survival, particularly in Staphylococcus aureus. Human evidence for these phenotypes is strongest in diabetes-related foot infection and associated bone, whereas evidence for intracellular Pseudomonas aeruginosa in wounds remains largely experimental. We examined whether chemically defined compounds associated with traditional Chinese medicine (TCM) might improve antibiotic performance in this setting. The most persuasive preclinical studies connect a compound to antibiotic entry, target regulation or viable killing. Weaker reports rely on early-biofilm biomass, checkerboard interaction or formulation effects that cannot be assigned to the phytochemical itself. Cinnamaldehyde, chenodeoxycholic acid, sodium houttuyfonate and 5, 8-dihydroxy-1, 4-naphthoquinone provide useful mechanistic or infected-wound leads, but the evidence remains fragmented across organisms and models. As of 12 August 2026, we found no controlled human study showing that a chemically defined TCM-associated compound used as an antibiotic adjunct clears mature chronic-wound biofilm, prevents infectious relapse or safely reduces antibiotic exposure. Progress now depends on testing fewer, well-defined combinations in mature polymicrobial systems. Such studies should measure post-treatment regrowth, infection-site exposure and tissue compatibility alongside bacterial killing.

## 1 Introduction

Chronic wounds offer bacteria a set of protected, spatially uneven habitats. Ischaemia, neuropathy, recurrent trauma and devitalised tissue encourage growth in aggregates embedded in microbial and host material. A meta-analysis of 24 studies (2, 666 lesions) estimated that biofilm was detectable in 68% of chronic wounds, although sampling and detection methods varied substantially (Coelho et al., 2025). Biopsy microscopy has also confirmed mono- and multispecies aggregates (Johani et al., 2017; Dilhari et al., 2021). These findings help explain why an antibiotic that appears active in routine testing may kill poorly in tissue. They do not, by themselves, diagnose infection. In diabetes-related foot disease, diagnosis remains clinical. An investigational adjunct would be added to the usual pathway: drainage or debridement when required, off-loading, perfusion assessment, informative sampling and pathogen-directed antibiotics (Senneville et al., 2023).

*S. aureus* and *P. aeruginosa* provide the clearest mechanistic axis for this review, although chronic wounds are often polymicrobial. This focus is intentionally selective. It does not cover anaerobes, enterococci, Enterobacterales, fungi or every community configuration. MRSA can combine β-lactam resistance with matrix production, small-colony variants (SCVs) and intracellular survival. *P. aeruginosa* readily exploits oxygen and nutrient gradients in wound-like aggregates. Their interaction is neither fixed nor uniformly cooperative. Spatial organisation and secreted metabolites can suppress one partner, induce SCV phenotypes or alter susceptibility to a particular antibiotic (Fazli et al., 2009; Ibberson et al., 2022; Khalid et al., 2023). Matched clinical isolates can coexist, whereas other pairings rapidly select SCV-like *S. aureus*, even without the alkyl-quinolone mechanism often invoked for this competition (Roberts et al., 2026). A single planktonic laboratory strain is therefore a poor proxy for a mixed wound community.

We use ‘TCM-associated’ as a provenance label for a characterised constituent, derivative or formulation-linked molecule linked to a medicinal material used in TCM. It does not imply chemical uniqueness or an established adjunct. This distinction matters in combination studies. Direct antibacterial activity can masquerade as potentiation; metabolic suppression can protect survivors; and an active carrier can contribute more than its named cargo. We therefore ask which defined candidates alter a specified cause of antibiotic failure at an exposure plausibly achievable in a chronic wound.

## 2 Biofilm-associated tolerance, persister-like survival and protected reservoirs in chronic wounds

### 2.1 Protected, spatially heterogeneous communities

Human-tissue imaging makes a simple surface-film model difficult to sustain. Peptide nucleic acid fluorescence *in situ* hybridisation and microscopy have identified matrix-associated microcolonies that routine culture did not fully capture (Kirketerp-Møller et al., 2008). In venous leg ulcers, *P. aeruginosa* aggregates lay deeper than *S. aureus* aggregates (Fazli et al., 2009). Diabetic-foot biopsies likewise show wide variation in aggregate size, depth, species composition and surrounding matrix (Johani et al., 2017; Dilhari et al., 2021). These findings matter for both sampling and treatment. A surface swab may miss a protected compartment. A locally applied drug may also fail to reach the tissue concentration predicted from broth activity.

Matrix impedes some agents, but it is only part of the explanation for poor killing. Extracellular polysaccharides, proteins, host material and extracellular DNA can bind or retard a drug. Other antibiotics traverse the aggregate yet remain ineffective because the cells they reach are growing slowly or have altered the targeted process. Aggregate size and spacing create gradients in oxygen, electron acceptors, pH, metabolites and antibiotic concentration. Stress responses, low anabolic activity and altered target expression then affect each drug class differently. Calling all of these effects ‘biofilm resistance’ hides the distinction between heritable resistance, reversible physiological tolerance, uneven exposure and a slowly dying survivor fraction.

Longitudinal sequencing suggests that community behaviour, rather than the presence of a single taxon, may influence outcome. Temporal stability, strain-level variation in *S. aureus* and metagenomic biofilm signatures have each been associated with healing (Loesche et al., 2017; Kalan et al., 2019). Composition and viable spatial burden are different measurements. Relative abundance may rise while absolute counts fall. In 117 venous leg ulcers treated with frequent debridement, a microscopy-derived biofilm metric did not predict eight-week healing (Stewart et al., 2024). The association is informative, but it is not universal.

Experiments show the same dependence on context. In a polymicrobial mouse wound, spatial organisation changed *S. aureus* susceptibility to aminoglycosides but not vancomycin (Ibberson et al., 2022). A five-species community produced treatment tolerance, spatial partitioning and SCV-like *S. aureus* in the presence of *P. aeruginosa* (Khalid et al., 2023). Activity that survives changes in strain pairing, community geometry and antibiotic partner is more persuasive than a result labelled simply ‘wound biofilm’. **Figure 1** summarises the wound niche, distinct survivor states and routes back to recurrence.

**Figure 1:**
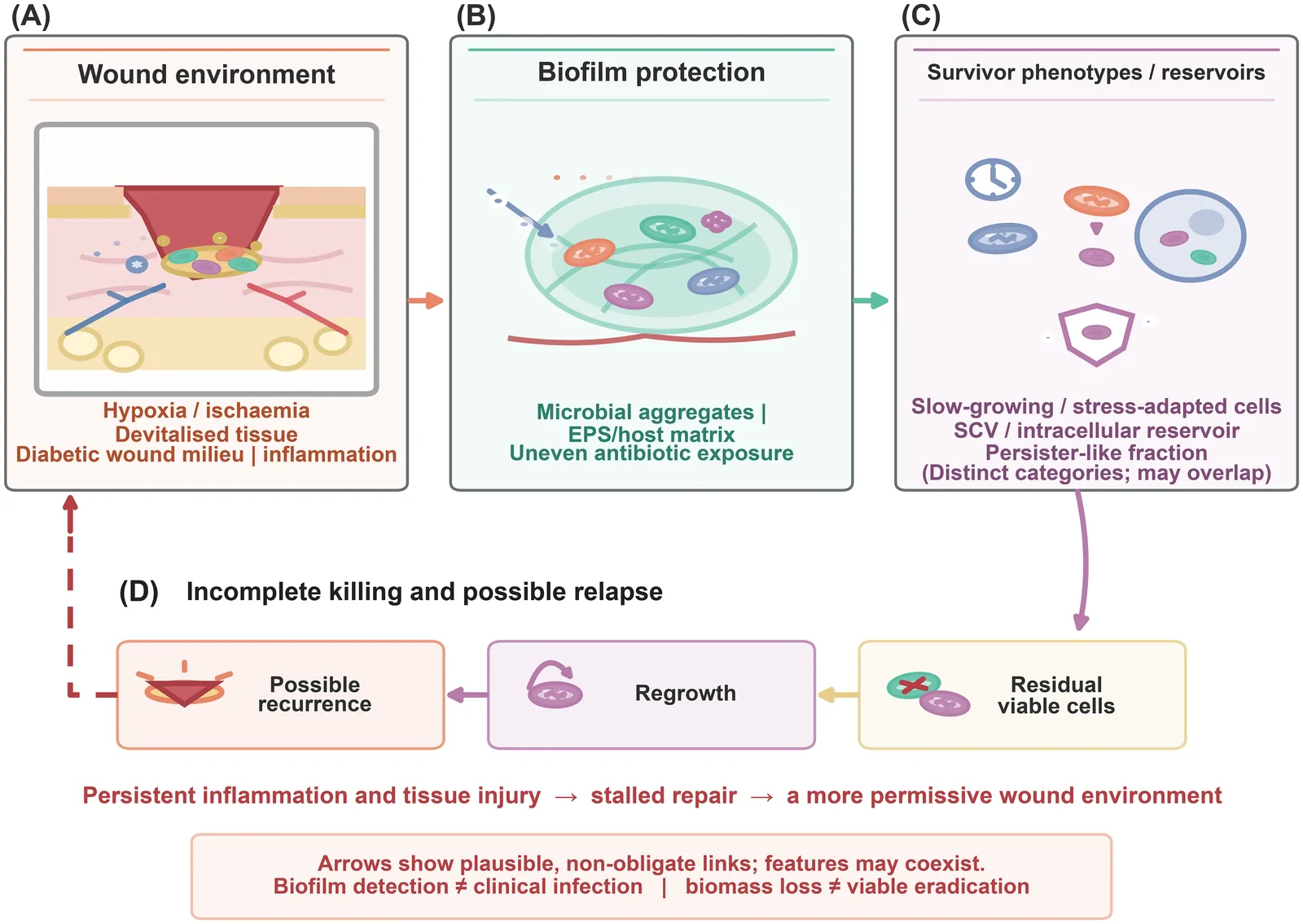
From a permissive wound environment to possible recurrence. **(A)** Hypoxia or ischaemia, devitalised tissue, the diabetic wound milieu and persistent inflammation may create conditions that favour aggregation. **(B)** Spatial microbial aggregates, bacterial and host matrix, local gradients and uneven antibiotic exposure can protect viable cells. **(C)** Slow-growing or stress-adapted cells, persister-like fractions, small-colony variants and intracellular reservoirs are distinct categories, although they may overlap. **(D)** Incomplete killing can leave viable cells that regrow after treatment withdrawal and may contribute to clinical recurrence. The clockwise arrows denote plausible, non-obligate links and feedback through impaired repair; these features may coexist or diverge. Biofilm detection alone does not establish clinical infection, and biomass reduction does not establish viable eradication. SCV, small-colony variant.

### 2.2 How the wound microenvironment shapes antibiotic tolerance

Of the microenvironmental pressures found in chronic wounds, oxygen limitation has the strongest mechanistic link to antibiotic tolerance. Microsensor and transcriptomic studies have demonstrated steep gradients in *in vitro* aggregates, diabetic mouse wound scabs and ex vivo human chronic-wound specimens, together with *P. aeruginosa* signatures consistent with oxygen depletion (James et al., 2016). Restricted oxygen and alternative electron acceptors in wound-fluid-like medium induced stress responses, curtailed biosynthesis and growth, and increased ciprofloxacin tolerance (Stewart et al., 2019). Dense, gel-entrapped *S. aureus* aggregates developed comparable oxygen limitation, heterogeneous gene expression and tolerance to several antibiotics (Pabst et al., 2016). Hypoxia is thus a credible driver, although no single gradient explains every recalcitrant wound.

The diabetic milieu can strengthen matrix production, but high glucose is not a synonym for bacterial dormancy. Advanced glycation end products promoted *S. aureus* biofilm formation *in vitro* through SigB upregulation, LrgA downregulation and greater extracellular-DNA release (Xie et al., 2020). In a five-species wound model, the biofilm was less metabolically active than planktonic culture. Yet a high glucose concentration increased rather than decreased metabolic activity, and SCV-like *S. aureus* colonies were associated instead with *P. aeruginosa* (Khalid et al., 2023). The culture data separate glucose availability from dormancy. In an intact wound, hyperglycaemia may still favour persistence through microvascular dysfunction, impaired neutrophil function and defective repair. Bacterial culture cannot reproduce these host processes.

Wound pH raises the same problem of cause and consequence. In an observational cohort of 99 patients with 106 diabetic foot ulcers, 76.4% of wounds were alkaline at baseline and pH fell as the wounds improved during standardised treatment (Wang et al., 2024b). Alkalinity can change bacterial surface charge, enzyme activity, antibiotic ionisation and proton motive force. Because bacterial metabolism and tissue breakdown can also alter pH, the cohort cannot tell whether alkalinity contributed to poor healing or simply accompanied it. For candidate screening, the practical lesson is narrower: activity measured only in neutral broth may not predict performance in an alkaline wound.

Redox biology is bidirectional. Iron-dependent oxidative stress can sustain pro-inflammatory macrophage activity, fibroblast senescence, protease activity and extracellular-matrix injury in a non-healing wound (Wlaschek et al., 2019). Excessive neutrophil extracellular traps are associated with impaired diabetic-wound healing, infection severity and amputation risk (Fadini et al., 2016; Yang et al., 2020). Localised oxidative killing nevertheless contributes to host defence and to several antibiotic-adjuvant combinations. A broad antioxidant could protect tissue while blunting bacterial killing. An oxidising treatment could lower burden at the cost of keratinocyte or fibroblast injury. Separate time courses for bacterial killing and repair reveal which effect dominates.

A layered wound-like system supports spatially organised coexistence of *S. aureus* and *P. aeruginosa* (Chen et al., 2021). An updated collagen-based model combines stable coexistence with a pH above 8 and steep oxygen gradients (Thaarup et al., 2023). Wound-like medium can produce rapid biofilm development and SCV-like colonies. In one study, it reduced virulence in a nematode assay (Pouget et al., 2022). Each model exposes a different weakness in a proposed mechanism. A signal that persists across wound-like medium and collagen is more persuasive than one confined to broth. Ex vivo skin and splinted diabetic, ischaemic or porcine wounds then test tissue association, repair and longer treatment courses.

### 2.3 Defining resistance, tolerance and protected survivor phenotypes

Resistance, tolerance and persistence describe different measurements. Resistance permits growth at a higher antibiotic concentration and is commonly reflected by a raised MIC. Tolerance lengthens the time required to kill most of a population without necessarily changing the MIC. A minimum duration for killing (MDK) provides one quantitative approach. Persistence is a heterogeneous form of tolerance. A subpopulation dies much more slowly than the majority, often producing a biphasic time–kill curve (Brauner et al., 2016, 2017; Balaban et al., 2019). These are operational definitions. Residence in a biofilm, a small colony, low ATP or survival at one sampling point is not enough to identify a persister.

An anti-persister experiment needs time-resolved killing at a bactericidal exposure and recovery after drug removal. It should also confirm that regrown descendants retain the parental MIC and reproduce the result across independent cultures (Balaban et al., 2019). Resistant mutants, drug decay, carry-over and incomplete antibiotic access need separate controls. Mature-biofilm work adds viable burden, architecture and regrowth. Crystal violet reports attached biomass. A metabolic signal may fall simply because cells have become quiescent. Neither endpoint establishes eradication on its own (Azeredo et al., 2017).

In diabetic foot disease, direct human evidence is stronger for SCVs and intracellular reservoirs than for rigorously defined persisters. *S. aureus* SCVs have been recovered from diabetes-related foot ulcers or infection. They have also been found in intracellular and macroscopically uninvolved bone compartments, providing a plausible route to relapse (Lee et al., 2023). Enrichment-based work in chronic-wound specimens has also shown why routine diagnostics can miss this slow-growing phenotype (Carris et al., 2025). SCVs remain colony phenotypes. They may reflect stable metabolic mutation or reversible regulation, but they are not a substitute for a killing curve. Another wound-isolate study found SCVs in only 1.1% of *S. aureus* isolates, underscoring the effects of cohort, sampling and prolonged culture (Min et al., 2022). Intracellular *S. aureus* in bone and other host cells may evade drugs with poor cellular penetration, although therapeutic evidence is still largely preclinical (Zelmer et al., 2022).

Intracellular *P. aeruginosa* aggregates have been visualised in host cells, including macrophages, in wounded zebrafish. Cell-permeable antibiotics performed better in this model, and aggregate-dispersing molecules partly restored susceptibility (Pont et al., 2025). Whether a comparable reservoir contributes to human chronic wounds remains unknown. We reserve ‘anti-persister’ for studies that demonstrate an appropriate killing curve, regrowth and retained susceptibility. We use ‘persister-like’ when the survivor state is suggestive but incompletely defined.

Together, these findings argue against a single biofilm endpoint. Matrix loosening, greater drug entry, killing of survivors and resolution of inflammation are related steps. Success at one does not guarantee the next. These steps organise the candidate literature below.

Review approach and compound selection. We searched PubMed for English-language publications available by 12 August 2026, prioritising work from January 2016 onwards; earlier studies were retained when they established a method, concept or clinical benchmark. We screened titles, abstracts and reference lists for chemically defined compounds or clearly characterised formulations tested with antibiotics against S. aureus, P. aeruginosa, A. baumannii or mixed wound-relevant communities in planktonic, biofilm, tolerance, small-colony variant, intracellular or infected-wound models. We retained studies reporting an antimicrobial endpoint, including MIC, FICI, MBIC or MBEC, viable CFU, time–kill, post-treatment regrowth or an *in vivo* infection outcome. Direct-antibacterial studies were retained as scope-boundary comparators when they informed attribution or wound relevance. We excluded uncharacterised crude extracts, docking-only reports, food-preservation studies and wound-healing studies without an antimicrobial endpoint. Because organisms, interventions and outcomes were heterogeneous, we used a critical narrative synthesis rather than pooled analysis.

## 3 Candidate mechanisms and the evidence behind them

The candidate literature is best read as a series of attempts to remove particular constraints on antibiotic action. Some compounds alter adhesion or quorum-regulated behaviour; others affect entry, energetics or the physiology of surviving cells. Few have been followed from a molecular observation into a wound model (Thaarup et al., 2022; Lee et al., 2023). **Table 1** reports mechanistic support and wound relevance separately.

**Table 1:** Representative TCM-associated compounds and scope-boundary comparators: mechanistic support and relevance to wound biofilms.

Candidate and source context	Antibiotic, organism and state	What the experiments showed	Wound-model evidence	Mechanistic support	Wound relevance tier	Limitation most likely to change interpretation	Representative reference(s)
Baicalein; flavone associated with *Scutellaria baicalensis*	Vancomycin, linezolid or ciprofloxacin; *S. aureus* or *P. aeruginosa* biofilm	Altered Agr/RNAIII/SarA, ica and QS readouts and improved viable antibiotic activity; wound benefit required a nanoparticle pair	Established biofilm models; ciprofloxacin–baicalein nanoparticles in an infected rat wound	M2 (orthogonal preclinical)	W2 (animal wound)	High concentrations and a carrier-dependent wound result; no chronic-wound exposure or recurrence study	(Chen et al., 2016; Liu et al., 2020a, 2023; Muniyasamy and Manjubala, 2024)
Baicalin; glycoside of baicalein associated with *S. baicalensis*	Levofloxacin, linezolid, cefotaxime or other tested antibiotics; *S. aureus* or *P. aeruginosa* biofilm	Reduced matrix and viable burden and altered Las/Rhl/Pqs-related signalling; oxacillin and cefotaxime studies added permeability measures, but docking was not target engagement	Mouse peritoneal implant and rat biofilm-associated infection models; neither was a cutaneous wound	M2 (orthogonal preclinical)	W0 (non-wound *in vivo* infection)	Chemistry and exposure differ from baicalein; no wound PK, relapse test or tissue-safety window	(Luo et al., 2017; Du et al., 2019, 2023; Meng et al., 2025, 2026)
Cinnamaldehyde; phenylpropanoid in *Cinnamomum cassia* volatile oil	Ampicillin or cefotaxime in clinical MRSA; gentamicin or colistin in separate carrier/biomass studies	SarA deletion/complementation linked β-lactam rescue to mecA/PBP2a; newer carrier and colistin studies added viable or biomass signals without comparable causality	Wound isolates; non-wound infection/biofilm models	M3 (causal preclinical)	W1 (wound isolates)	The causal SarA chain has not reached a chronic wound; carrier and biomass results cannot inherit its M3 level	(Li et al., 2024; Ahsan et al., 2026; Çalı et al., 2026)
Berberine; isoquinoline alkaloid in *Coptis* and *Phellodendron* species	Gentamicin with blue light; also clindamycin, rifampicin or imipenem; MRSA or *P. aeruginosa* biofilm	Context-dependent membrane, energetic and expression effects; repeated photoactivated cycles reduced viable counts and regrowth	Mature biofilm using diabetic-foot isolates; no *in vivo* test of the photoactivated antibiotic pair	M2 (orthogonal preclinical)	W1 (wound isolates)	Activity varies by isolate, antibiotic, pH and illumination; the photoactivated series lacks independent replication	(Su and Wang, 2018; Xia et al., 2022; Zhang et al., 2022; Zhao et al., 2023b; Seo et al., 2024; Zhou et al., 2024; Zeraatpisheh et al., 2025; Gonçalves et al., 2026a, 2026, 2026)
Chenodeoxycholic acid; endogenous mammalian bile acid detected through Tanreqing-related screening	Amikacin; MRSA, SCVs, biofilm-associated cells and antibiotic-enriched survivors	PMF-dependent aminoglycoside uptake and weaker superoxide-dismutase defence accompanied time-dependent killing across difficult phenotypes	Murine non-wound infection only	M3 (causal preclinical)	W0 (no cutaneous-wound context)	Not chemically unique to TCM; no diabetic/ischaemic wound, local PK, repeated-dose safety or amikacin-sparing test	(Cui et al., 2023)
Curcumin; diarylheptanoid associated with *Curcuma longa*	Colistin against *A. baumannii* survivors; separate moxifloxacin or azithromycin wound formulations	The colistin pair had ROS/permeability evidence; the wound studies evaluated multi-component sequential or pH-responsive formulations	The formulations, not the free colistin pair, were tested in infected mouse models	M2 for the colistin pair	Free colistin pair W0; separate formulations W2	Wound activity cannot be assigned to free curcumin without complete *in vivo* factorial controls	(Kaur et al., 2018; Qiao et al., 2024; Zhang et al., 2025)
Bakuchiol; meroterpene associated with *Psoralea corylifolia*	Colistin; *A. baumannii* antibiotic-enriched survivors; direct activity against *S. aureus* survivors	Membrane studies supported complementary disruption of phospholipid-rich and lipooligosaccharide regions	No wound model	M2 (orthogonal preclinical)	W0 (no cutaneous-wound context)	*In vitro* only; incomplete consensus persistence controls and no tissue exposure, recurrence model or *in vivo* tolerability	(Suh et al., 2025)
Sodium houttuyfonate; defined bisulfite adduct derived from houttuynin of *Houttuynia cordata*	Penicillin G against clinical MRSA; meropenem against heteroresistant *P. aeruginosa*	The penicillin pair interacted across 20 MRSA isolates and improved wound closure and inflammatory histology; the meropenem pair added time–kill, early-biofilm and motility data	MRSA-infected rat wound for the penicillin pair; no wound model for the meropenem pair	M1 (interaction signal; no quantitative wound infection-control endpoint)	W2 for penicillin pair; W0 for meropenem pair	The acute wound study lacked tissue CFU, mature-biofilm viability and a validated causal target	(Li et al., 2025a, 2025)
5, 8-dihydroxy-1, 4-naphthoquinone (DHNQ); naphthazarin/shikonin-pharmacophore analogue	Gentamicin; MRSA biofilm and infected wound	Membrane and metabolic changes accompanied a combination-related reduction of about 4 log10 CFU	MRSA-infected mouse wound; keratinocyte, fibroblast and haemolysis screens	M2 (orthogonal preclinical)	W2 (animal wound)	No chronic diabetic/ischaemic phenotype, local exposure or post-withdrawal recurrence	(Han et al., 2026)
Honokiol; biphenolic lignan associated with *Magnolia* species	Oxacillin in an older MRSA interaction study; direct honokiol in nutrient-stressed MRSA	Older antibiotic potentiation was followed by direct hexose-phosphate transport regulator A-linked nutrient and redox effects in a separate study	Direct honokiol, not the oxacillin pair, was tested in an MRSA-infected mouse wound	M1 for adjunct pair; M3 for separate direct activity	Adjunct-pair W0; separate direct-activity W2	The direct wound result does not validate rescue of oxacillin	(Kim et al., 2015; Wei et al., 2026)
Carvacrol; monoterpene phenol present in *Mosla chinensis* and other plants	Ciprofloxacin plus blue light; four-species mature biofilm	The complete light–carvacrol–ciprofloxacin regimen reduced viable polymicrobial biofilm; a separate light–carvacrol treatment reduced burden in murine burn wounds	No wound test of the antibiotic-containing regimen; separate antibiotic-free burn-wound platform	Adjunct platform M2	Antibiotic platform W0; separate direct platform W2	The burn-wound result cannot be transferred to the ciprofloxacin-containing regimen	(Li and Wu, 2021; Lu et al., 2021; Zheng et al., 2021)

M and W record the highest claim-specific level reached for the named pair or platform; they are review-specific descriptive labels, not validated quality or risk-of-bias scores, and cannot be transferred to another antibiotic, formulation or photoactivation regimen. M1, screening or interaction signal; M2, viable killing or mature-biofilm effect supported by an independent mechanistic measure; M3, causal target or uptake evidence plus *in vivo* infection validation; M4, controlled human anti-infective evidence. W0, no cutaneous-wound context; W1, wound isolate or wound-like model; W2, infected-wound animal model; W3, infected human wound. No candidate has reached M4 or W3 for an antibiotic-adjunct claim. ‘TCM-associated’ describes provenance, not chemical exclusivity, validation by historical use or established adjunct status. CFU, colony-forming units; MRSA, methicillin-resistant *Staphylococcus aureus*; PK, pharmacokinetics; PMF, proton motive force; QS, quorum sensing; ROS, reactive oxygen species; SCV, small-colony variant; TCM, traditional Chinese medicine.

M and W are claim-specific descriptive labels used in this review; they are not validated quality or risk-of-bias scores. M1 denotes a screening or interaction signal. M2 requires viable killing or a mature-biofilm effect supported by an independent mechanistic measure. M3 requires causal target or uptake evidence together with *in vivo* infection validation. M4 requires controlled human anti-infective evidence. W0 indicates no cutaneous-wound context, W1 a wound isolate or wound-like model, W2 an infected-wound animal model and W3 an infected human wound. The highest level is assigned only to the named compound-antibiotic pair or complete platform. Cinnamaldehyde is M3 for the SarA-linked β-lactam pair because deletion/complementation, target-linked changes and murine infection validation converge; its separate carrier and colistin studies do not inherit that level. Baicalein is M2 because viable activity is supported by independent pathway or permeability findings, but causal target validation and *in vivo* validation for the same free-compound pair are lacking. Sodium houttuyfonate illustrates a different boundary: wound closure and histology support W2, but not M2 or M3 without tissue CFU, mature-biofilm viability or a validated mechanism. The same labels and compound names are used in the main text and tables.

### 3.1 Modulation of adhesion, quorum sensing and extracellular matrix

Most adhesion studies begin treatment at inoculation and test early development rather than established biofilm. For a chronic-wound question, the informative extension is viable killing in a mature, protein-rich mixed community (Fanaei Pirlar et al., 2020).

Baicalein and baicalin illustrate both the promise and the interpretive difficulty. Baicalein altered *agrA*/RNAIII, *sarA* and *ica*-associated expression in *S. aureus* and improved vancomycin activity in three- and seven-day biofilms (Chen et al., 2016); a rifampicin study added microscopy of established biofilm but no withdrawal/regrowth test (Muniyasamy and Manjubala, 2024), and baicalein also suppressed quorum-regulated behaviour in *P. aeruginosa* (Luo et al., 2016). Baicalin, the glycoside, reduced *S. aureus* matrix and viable burden and enhanced levofloxacin or linezolid activity (Du et al., 2019, 2023); interaction with oxacillin was later linked to permeability and early-biofilm measures (Meng et al., 2025).

In *P. aeruginosa*, baicalin influenced Las, Rhl and Pqs signalling and improved antibiotic-mediated clearance in a mouse implant model (Luo et al., 2017); a later cefotaxime study added permeability, viable-biofilm and metabolomic measurements (Meng et al., 2026). The docking result in that study remains a proposed target rather than target engagement, and a separate single-strain baicalein–azithromycin screen added little causal information (Parra Rodríguez et al., 2026). Because glycosylation changes exposure and potency, the two compounds are evaluated separately.

Quercetin and bakuchiol have also inhibited Las/Rhl-dependent behaviour, and biophysical work supports interference by bakuchiol with LasR dimer function (Ouyang et al., 2016; Alasiri et al., 2024). Such experiments identify an antivirulence mechanism; they say little about viable clearance of a mature wound biofilm. Altering quorum sensing may change dispersal and community composition in either direction, depending on dose, timing and the antibiotic partner.

Emodin offers a matrix-directed example. At sub-inhibitory exposure it reduced extracellular-DNA release and altered several regulatory and matrix-associated genes in *S. aureus* (Yan et al., 2017). An aptamer-targeted tetrahedral nucleic-acid carrier later lowered MRSA burden, changed biofilm readouts and improved repair in murine skin infection (Jiang et al., 2026). The carrier supplied targeting and exposure advantages not demonstrated for free emodin, so the result belongs to the formulated intervention rather than to emodin alone.

Quorum-sensing modulation is not predictably antibiofilm. At sub-inhibitory concentrations, berberine suppressed *S. aureus* AgrA, inhibited dispersal and increased aggregation and *icaA*-associated matrix production (Zhang et al., 2022). Emodin shows a similar dependence on the partner and endpoint: recent work reported improved cefalexin control of adhesion and invasion and better amoxicillin activity in macrophages, whereas another study found antagonism with ampicillin in established MRSA biofilm (Chu et al., 2025; Zhao et al., 2025). Dose, genotype, growth phase and treatment sequence determine which interpretation is justified.

### 3.2 Drug access, bacterial energetics and target-linked antibiotic killing

After an antibiotic enters an aggregate, killing may still fail because permeability is restricted, efflux is active, metabolism is low or the target is poorly engaged. MIC shifts and efflux-gene expression are useful leads, but stronger studies connect these findings to drug uptake or viable killing.

Cinnamaldehyde provides the clearest target-linked evidence in wound-derived isolates. It potentiated ampicillin and cefotaxime in 18 clinical MRSA isolates, seven from wound exudate, and improved mature-biofilm killing. Deletion and complementation placed SarA upstream: greater SarA phosphorylation reduced promoter binding, *mecA* expression and penicillin-binding protein 2a abundance (Li et al., 2024). Later studies were less conclusive: a gentamicin co-delivery system reduced viable counts in mature PAO1 biofilm only within an active liquid-crystalline carrier (Ahsan et al., 2026); cinnamaldehyde or eugenol was synergistic with colistin in only half of 10 resistant *A. baumannii* isolates, with biomass but no viable-clearance endpoint (Çalı et al., 2026). The SarA study remains the strongest causal chain.

Berberine has a larger, less consistent literature. Planktonic MRSA shows membrane leakage and lipid changes (Zhang et al., 2020), while combinations with clindamycin, rifampicin and imipenem have shown activity (Su and Wang, 2018; Xia et al., 2022). Yet lower *mexZ*, *mexX*, *mexY* and *oprM* expression accompanied greater intracellular berberine without resolving the efflux direction (Su and Wang, 2018), and Corydalis/berberine work relied on checkerboards and indirect ethidium-bromide measurements (Seo et al., 2024). Synergy with amikacin occurred in only 6 of 50 MRSA strains (Zhou et al., 2024); a ciprofloxacin combination active against PAO1 neither cleared preformed biofilm nor reproduced reliably in carbapenem-resistant clinical isolates (Zeraatpisheh et al., 2025). Proton-motive-force effects at pH 8.5–9.0 in synovial-fluid aggregates may not transfer to wound fluid (Zhao et al., 2023b). The evidence fits a context-dependent membrane or energetic modulator, not a validated efflux inhibitor.

Other access-related candidates remain exploratory. Baicalein increased ethidium-bromide accumulation but had modest time–kill effects (Moulick and Roy, 2024); a tanshinone I derivative increased intracellular antibiotic accumulation and improved streptomycin or ampicillin activity outside a wound model (Wang et al., 2017). Astrakurkurone, a natural-product comparator outside the core TCM-associated set, paired cefoxitin interaction with β-lactamase, membrane and ROS measurements and reduced survivors on several ex vivo substrates, although active concentrations were high and persistence controls incomplete (Bandyopadhyay et al., 2026). Honokiol raises a different attribution issue: older work examined oxacillin interaction, whereas an infected-wound study tested direct activity under nutrient stress and linked it to hexose-phosphate transport protein A (HptA) (Kim et al., 2015; Wei et al., 2026). The latter supports honokiol as an antibacterial lead while leaving the oxacillin question open.

Chenodeoxycholic acid with amikacin has a more coherent chain. By increasing aminoglycoside uptake through a proton-motive-force-dependent process and weakening superoxide-dismutase defence, the pair killed conventional MRSA, biofilm-associated cells, SCVs and antibiotic-enriched survivors and was active in a murine infection model (Cui et al., 2023). The experiments went beyond inference from biofilm residence, although biphasic killing, susceptible regrowth and rechallenge were incomplete. Translation now turns on tissue exposure and repeat-dose tolerance. As chenodeoxycholic acid is an endogenous mammalian bile acid identified through Tanreqing-related screening, the data apply to the molecule, not the parent formulation or a TCM-specific chemical class.

Sodium houttuyfonate reaches an infected-wound model, but with a less complete mechanism. This bisulfite-adduct derivative of houttuynin, associated with *Houttuynia cordata*, had a fractional inhibitory concentration index of 0.375–0.5625 with penicillin G across 20 clinical MRSA isolates: 14 met the study’s synergy threshold and six were additive (Li et al., 2025a). In an acute rat wound the combination improved closure and inflammatory histology, yet tissue CFU and mature-biofilm viability were not measured and the network-pharmacology/docking mechanism remained unvalidated. It also enhanced meropenem killing and reduced early-biofilm and motility measures in heteroresistant *P. aeruginosa*, at high concentrations and without a wound test (Li et al., 2025b). It remains one of few wound-tested pairs, with a less complete evidence chain than cinnamaldehyde or DHNQ–gentamicin.

The 5, 8-dihydroxy-1, 4-naphthoquinone (DHNQ)–gentamicin pair gives a more informative infected-wound result. DHNQ is a naphthazarin/shikonin-pharmacophore analogue from a screen of shikonin-related compounds, not a confirmed natural constituent of *Arnebia*. In MRSA it altered membrane and metabolic measures, reduced viable burden by about 4 log10 colony-forming units (CFU), improved healing in mice and passed preliminary keratinocyte, fibroblast and haemolysis screens (Han et al., 2026). This stronger viable-killing and tissue-compatibility evidence makes local pharmacokinetics and durability after withdrawal the immediate questions.

Energetic manipulation can also work against the antibiotic. A greater proton motive force may improve aminoglycoside entry, whereas quercetin-induced ATP depletion increased antibiotic-surviving *S. aureus* in a timing-dependent manner (Kim and Kim, 2025). Changes in membrane potential, reactive oxygen species (ROS) or ATP support sensitisation only when the same dosing sequence improves viable killing.

### 3.3 Candidate activity against SCVs, persister-like cells and intracellular reservoirs

Evidence is thinner when the intended target is an SCV, persister or intracellular reservoir. Chenodeoxycholic acid–amikacin tested SCVs, biofilm-associated cells and antibiotic-enriched survivors rather than inferring persistence from biofilm residence (Cui et al., 2023). Curcumin reduced the survivor fraction after colistin exposure in *Acinetobacter baumannii*, with antioxidant rescue, permeability and transcriptional data pointing to an ROS-linked mechanism (Kaur et al., 2018). Bakuchiol was active against enriched *S. aureus* and *A. baumannii* survivor populations and disrupted complementary membrane regions (Suh et al., 2025). These experiments did not reach a chronic-wound model or demonstrate recurrence prevention after withdrawal. The opposite quercetin results across *P. aeruginosa* quorum sensing and *S. aureus* energetic stress further show that organism and treatment order cannot be inferred from a compound name alone (Ouyang et al., 2016; Kim and Kim, 2025).

Intracellular delivery poses a separate problem. Liposomal berberine and curcumin reduced MRSA biofilm formation and intracellular infection in macrophage culture, whereas the free compounds did not show the same interaction (Bhatia et al., 2021). Dual-targeted nanoparticles carrying vancomycin and curcumin likewise improved access to macrophages and intracellular MRSA in cell and animal models (Zhao et al., 2023a). These carrier-enabled results point to intracellular viable burden over time as the relevant measure, alongside host-cell toxicity and recurrence after treatment withdrawal; they do not establish an intrinsic anti-persister effect of the free compounds.

### 3.4 Formulation- and photoactivation-dependent effects

Photoactivation changes the nature of the intervention. Repeated blue-light treatment with berberine or curcumin plus gentamicin reduced viable counts and post-treatment regrowth in preformed staphylococcal and dual-species biofilms, including diabetic-foot isolates; a related *P. aeruginosa* protocol incorporated sub-inhibitory colistin (Gonçalves et al., 2024, 2025, 2026, 2026, 2026). Carvacrol, a monoterpene phenol found in the TCM-associated herb *Mosla chinensis* but not unique to it, provides an earlier comparator: blue light plus carvacrol and ciprofloxacin reduced a mature four-species biofilm, while a separate light–carvacrol regimen reduced burden in murine burn wounds without an antibiotic partner (Li and Wu, 2021; Lu et al., 2021; Zheng et al., 2021). These results belong to the complete light-activated treatment. A small human study illustrates why the light component cannot be treated as inert: blue light alone reduced bacterial counts in 22 chronic wounds immediately after exposure, but the effect had disappeared at follow-up and healing did not differ by dose (Plum et al., 2026). Future work will have to resolve light penetration, oxygen dependence, sensitiser exposure and cumulative tissue toxicity.

Recent materials studies provide useful boundary comparators. A metformin-based cationic covalent-organic framework produced photocatalytic reactive oxygen species and broad pathogen inactivation in a standalone material system (Liu et al., 2026). A hot-pressed metal-covalent organic-framework film achieved sunlight-driven disinfection and could be reused (Wang et al., 2024a). Neither study tested an antibiotic pair in a chronic wound. In an infected-wound model, a bacteria-targeted, photo-responsive Ag-MOF/berberine gel combined visible-light photodynamic activity, targeted binding and berberine release. That result belongs to the complete formulation; it does not establish free-berberine activity or antibiotic rescue (He et al., 2024).

For formulation and photoactivation studies, the minimum attribution set should include the free compound alone, antibiotic alone, empty carrier, compound-loaded carrier, vehicle, light-only or sham-light exposure, dark controls and the complete combination; heat-only and oxygen-matched controls are added when those variables may contribute. Viable CFU should be reported alongside biofilm architecture and post-washout regrowth, with measured release, tissue exposure and cytotoxicity. Without these comparisons, activity belongs to the complete platform and cannot be assigned to the free compound or to antibiotic potentiation. **Table 2** provides this control set as a reusable benchmark.

**Table 2:** Evidence needed to support common claims in chronic-wound adjunct research.

Proposed claim	Evidence needed	Wound-relevant test	Controls that matter	Claim supported	What the experiment does not show
Antibiotic potentiation or resistance reversal	Full concentration matrix and time–kill data; MICs for both agents; relevant clinical isolates	Causal resistance determinant or target engagement, with viable clearance at an achievable wound exposure	Both monotherapies, vehicle and, where possible, determinant-positive and -negative isogenic strains	Potentiated antibiotic activity; determinant-linked resistance reversal if causality is shown	One FICI near 0.5, growth delay, docking or lower resistance-gene mRNA alone
Prevention of biofilm formation	Exposure begins before or at inoculation; biomass plus viable count and an attachment or early-matrix endpoint	Clinical wound isolates under wound-like medium, pH and oxygen conditions	Growth-matched exposure, vehicle and each monotherapy	Reduced attachment or inhibited biofilm formation	Prevention is not eradication of a mature biofilm
Mature-biofilm burden reduction or clearance	Preformed biofilm; CFU plus architecture or matrix; stated exposure, limit of detection and recovery method	Mature polymicrobial collagen or ex vivo skin biofilm, followed by washout and regrowth after drug withdrawal	Both monotherapies, untreated and carrier controls, and neutralisation/carry-over control	Reduced viable burden; clearance only if viable counts remain below detection and recovery/regrowth is negative	A crystal-violet or metabolic-signal reduction alone is not eradication
Suppression of tolerance	MIC separated from killing kinetics; complete time–kill curve and an MDK-type measure in a defined physiological state	Hypoxic, diabetic-chemistry, wound-fluid or aggregate model followed by recovery	Equal inoculum, drug stability, active-exposure verification and a susceptible comparator	Accelerated killing or shortened MDK	Faster early killing or lower biomass without duration metrics
Anti-persister activity	Reproducible biphasic killing; survivor recovery; unchanged MIC after regrowth; rechallenge	Independent cultures and recurrence after withdrawal in mature biofilm or an infected-wound model	Resistant-mutant exclusion, drug carry-over/decay control and both monotherapies	Depletion of a defined persister population; otherwise, persister-targeting potential	One high-dose survivor count, low ATP, SCV morphology or biofilm residence
SCV or intracellular-reservoir activity	Phenotypic/genetic SCV characterisation or intracellular CFU with an extracellular-killing control	Relevant host cells, ex vivo tissue or infection model with cellular exposure and recurrence after withdrawal	Parent strain, extracellular and intracellular compartments, cytotoxicity and penetration controls	Activity against SCVs or reduced intracellular burden	An SCV or intracellular bacterium is not automatically a persister
Formulation-enabled or photoactivated activity	Factorial design separating compound, carrier or light, antibiotic and their combinations; measured release and exposure	Protein-rich mature biofilm or tissue with realistic light/oxygen conditions and repeated-dose recovery	Free compound, empty carrier, light-only/sham, each monotherapy and cytotoxicity controls	Activity of the defined formulated or photoactivated platform	The result cannot be assigned to the free phytochemical when the carrier or light is active
Host-microenvironment or repair benefit	Bacterial burden measured separately from inflammation, angiogenesis, collagen and closure	Splinted diabetic or ischaemic infected wound with tissue compatibility and histology	Uninfected wound, infected untreated, vehicle/carrier and both monotherapies	Improved repair while reducing bacterial burden	Lower cytokines, ROS or wound area alone is not anti-infective efficacy
Clinical infection-control benefit	Randomised adjunct-versus-vehicle comparison on identical standard care and antibiotic background	Clinical resolution and recurrence, surgery/amputation, harms and durable closure	Blinded assessment and stratification by infection severity, perfusion, organism and debridement	Improved infection outcomes when the adjunct is added to standard treatment	MIC, FICI, biomarkers, four-week area reduction or an uncontrolled healing study
Clinical antibiotic sparing	Randomised design with a prespecified reduction in antibiotic dose or duration and a non-inferiority or superiority margin	Clinical resolution and recurrence, total antibiotic exposure, resistance, rescue therapy, surgery/amputation, harms and durable closure	Matched wound care; blinded assessment; explicit rescue criteria; stratification by severity, perfusion, organism and debridement	Lower antibiotic exposure without clinically important loss of infection control or excess relapse/resistance	An add-on trial with identical antibiotic exposure in both groups cannot establish sparing

Each row distinguishes an informative screen from an experiment capable of supporting the stated claim. CFU, colony-forming units; FICI, fractional inhibitory concentration index; MDK, minimum duration for killing; MIC, minimum inhibitory concentration; SCV, small-colony variant.

High-resolution mass spectrometry identified 52 components in Shuganning injection, which showed partial interaction with linezolid, inhibited MRSA biofilm formation and improved outcomes in *Galleria mellonella* and a murine skin-infection model (Wang et al., 2026). Because no active constituent or combination was isolated, the study supports standardised formulation-level investigation but cannot be assigned to one defined antibiotic adjunct.

## 4 What translation to chronic-wound infection would require

### 4.1 Where an adjunct would sit in clinical care

Clinical use would begin from the same diagnosis and treatment pathway as any other wound intervention. Microbial detection alone does not establish infection, and antibiotics are not used simply to hasten closure of an uninfected diabetic ulcer (Swanson et al., 2022; Senneville et al., 2023). Deep or severe infection, gangrene, necrotising disease and severe ischaemia call for urgent surgical and, where appropriate, vascular assessment (Senneville et al., 2023). In less acute infection, debridement or drainage, off-loading, perfusion assessment, deep-tissue sampling when indicated and susceptibility-directed therapy define the background against which an adjunct could be judged. Debridement remains foundational even though its microbiological effects vary with sampling and time: an immediate before-and-after study detected little change in the surface community, whereas longitudinal metagenomics associated debridement-related shifts with better outcomes (Kalan et al., 2019; Verbanic et al., 2020). Trial protocols need to standardise this background care.

Topical guidance is less uniform. Wound-care consensus allows a time-limited topical antimicrobial approach with cleansing, debridement and reassessment, whereas the diabetic-foot guideline advises against several routine topical strategies because outcome data are insufficient (Swanson et al., 2022; Senneville et al., 2023). Reviews of chronic-wound antiseptics and topical antibiofilm modalities find few comparable trials and no secure class effect (Barrigah-Benissan et al., 2022; Astrada et al., 2026). Diagnosis is another weak point: a 2026 systematic review found no validated non-invasive scale designed specifically to diagnose wound biofilm (González-Suárez et al., 2026). For a TCM-associated candidate adjunct, that uncertainty favours a standardised, time-limited add-on study with prespecified stopping rules, tissue sampling and a credible local-exposure rationale. **Figure 2** places standard care above the experimental framework because clinically indicated source control and antibiotics proceed according to need throughout development.

**Figure 2:**
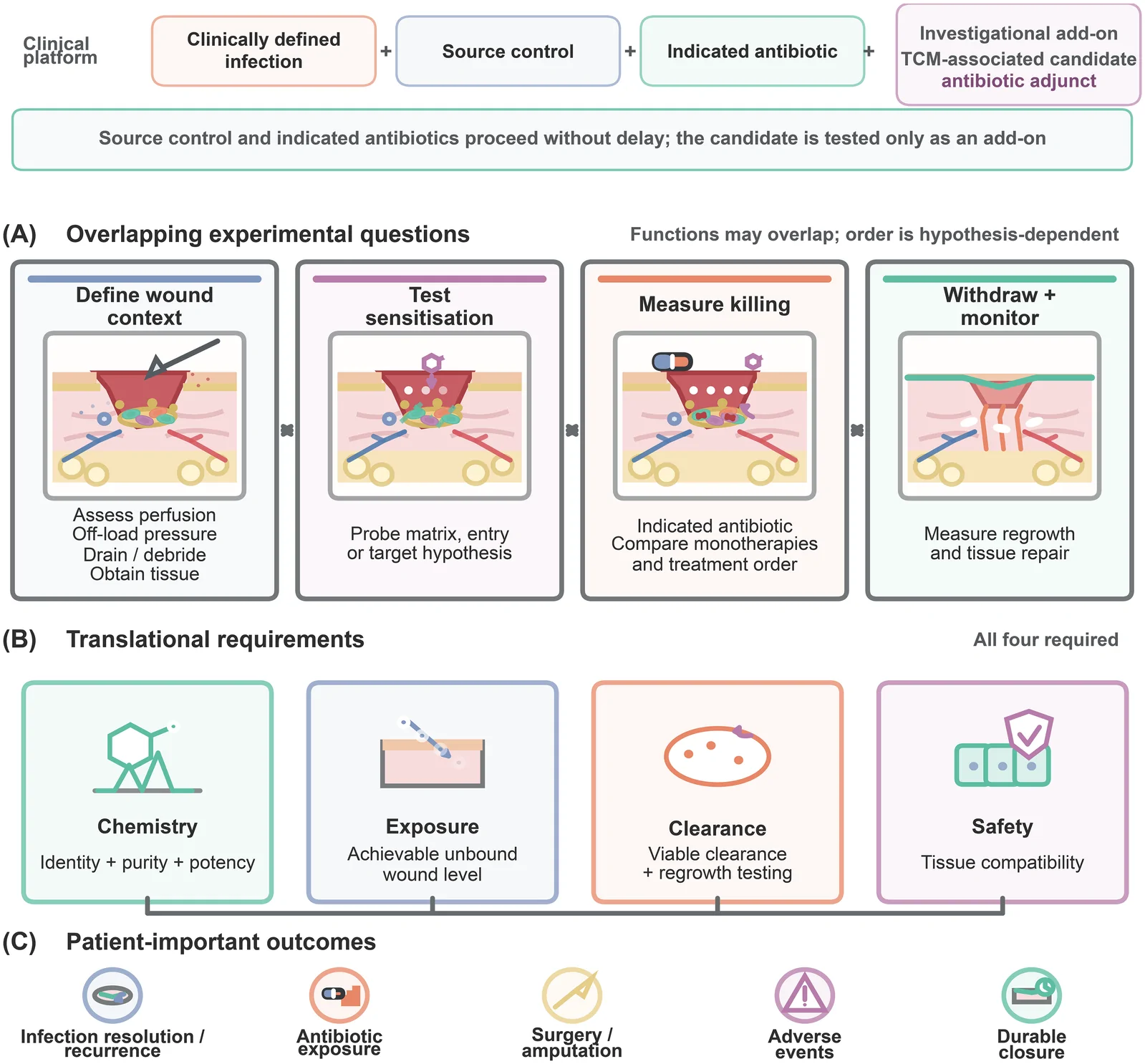
Translational framework for evaluating a TCM-associated compound as a candidate antibiotic adjunct in chronic wounds. Standard care proceeds according to clinical need and is not delayed by the investigational pathway; the candidate is evaluated only as an add-on to source control and indicated antibiotics. **(A)** Wound context, sensitisation, antibiotic killing and recovery after treatment withdrawal are overlapping experimental questions. Studies compare each agent alone, concurrent treatment and both treatment sequences, and assess viable killing, post-washout regrowth and repair. **(B)** Translation requires all four of the following: a chemically defined intervention, achievable unbound wound exposure, viable clearance with regrowth testing, and compatibility with repairing tissue. **(C)** Patient-important outcomes are infection resolution or recurrence, antibiotic exposure, surgery or amputation, adverse events and durable closure. TCM, traditional Chinese medicine.

### 4.2 Treatment order as an experimental variable

Treatment order is part of the mechanism. Matrix disruption before effective antibiotic exposure may release viable cells; an intervention intended to reduce antibiotic use has to be studied at the proposed lower dose or shorter course. **Figure 2A** treats wound context, sensitisation, antibiotic killing and recovery after treatment withdrawal as overlapping experimental questions whose order depends on the hypothesis. Mature-biofilm studies should include each monotherapy, simultaneous and sequential combinations, and regrowth after both agents are withdrawn.

### 4.3 Formulation, local delivery and attribution of effect

Local delivery may solve a genuine exposure problem, but it also creates a new intervention. Ciprofloxacin–baicalein nanoparticles improved bacterial and repair outcomes in a rat *P. aeruginosa* wound (Liu et al., 2023). By contrast, a sequential MRSA-wound hydrogel released moxifloxacin first and curcumin later as an anti-inflammatory cargo; that design did not test whether curcumin potentiated moxifloxacin (Qiao et al., 2024). Attribution depends on separating the free compound, carrier and antibiotic.

A recent review of TCM-based hydrogels describes hierarchical assembly across haemostasis, inflammation, proliferation and remodelling, while highlighting the attribution problems created by multi-component carriers (Ren et al., 2026). Its formulation-level framework does not establish antibiotic potentiation; each active component and carrier still requires factorial attribution.

Several newer nanocarrier, redox-active, photothermal and microneedle systems have reached infected-wound models (Shu et al., 2022; Li et al., 2023; Chen et al., 2026; Dong et al., 2026; Li et al., 2026a, 2026, 2026; Lu et al., 2026; Shen et al., 2026). Antibiotic-containing examples include a pH-responsive azithromycin–curcumin micelle and a mupirocin–piperine microemulsion hydrogel (Lin et al., 2025; Zhang et al., 2025). Because carrier behaviour, direct killing, heat, redox control and immune modulation can all contribute—and because the animal studies did not always include both antibiotic-only and phytochemical-only groups—these experiments evaluate the complete formulation. A curcumin–berberine ointment study presents the same attribution problem (Biernat et al., 2026).

A fucoidan–gentamicin–imipenem regimen reduced viable burden in a dual-species diabetic-rat wound (Nazari et al., 2026). We treat it as a scope-boundary study rather than a core TCM-associated candidate: the commercial fucoidan was reported only as a generic brown-seaweed polysaccharide, without a source species, molecular-mass distribution or sulphation profile. The absence of a fucoidan-only group and the unequal imipenem dose between comparator regimens also prevent the additional effect from being assigned to a reproducible polysaccharide preparation.

A multifunctional regimen must lower viable burden at a tissue-compatible exposure and still permit inflammatory resolution and durable re-epithelialisation. A strongly oxidising or membrane-active formulation may reduce bacteria while injuring keratinocytes, fibroblasts, endothelial cells or immune cells, whereas a fall in TNF-α, NF-κB activity, oedema or wound area may occur without infection control. Measuring bacterial and repair outcomes on the same time course makes that trade-off visible.

## 5 Discussion: a practical development agenda

### 5.1 Candidates, not a clinical class

Preclinical work now extends from diabetic-foot isolates and repeated regrowth assays to several infected-wound combinations (Li et al., 2025a; Gonçalves et al., 2026a, 2026, 2026; Han et al., 2026), but human anti-infective efficacy has yet to be tested. Variation in organism, partner drug, growth state and formulation prevents a class-level conclusion. The literature identifies individual candidates rather than ‘TCM’ as a wound-biofilm treatment.

Several barriers separate an encouraging laboratory pair from a clinical adjunct. Protein binding, tissue penetration, oxygen and pH gradients, debridement and variation in background care can all separate the nominal dose from the unbound exposure at the infected compartment. Polymicrobial communities and distinct wound phenotypes also limit generalisability, while a carrier or light regimen may add effects that are absent from the free-compound experiment. Translation therefore requires standardised source control, an exposure rationale, post-treatment recurrence endpoints and a randomised adjunct trial against matched standard care.

Human botanical studies answer a different question. ON101 improved healing of Wagner grade 1–2 ulcers (Huang et al., 2021); NF3 yielded mainly feasibility and exploratory inflammatory findings (Ko et al., 2014); and studies of Cortex *Phellodendri* fluid and Yunnan Baiyao reported healing outcomes (Liu et al., 2020b; Zhang et al., 2026). A small open-label randomised study of polygalacturonic–caprylic acid ointment versus medical-grade honey also reported six-week healing in 26 chronic wounds (Dagher et al., 2026). In diabetic mice, quercetin or rutin plus gentamicin was evaluated in a biofilm-seeded *P. aeruginosa* wound, but the interaction was non-synergistic and the combination was not clearly superior to gentamicin; a later correction replaced an erroneous histology image without changing the authors’ conclusion (Almuhanna et al., 2024, 2025). These studies concern repair or topical wound management and do not establish clinical antibiotic sparing or mature-biofilm clearance.

### 5.2 Let the claim determine the experiment

Two counterexamples explain the design of **Table 2**. Sub-inhibitory berberine reduced dispersal yet stabilised *S. aureus* aggregates, while quercetin-induced ATP depletion increased antibiotic survivors (Zhang et al., 2022; Kim and Kim, 2025). Neither result would be apparent from a single pathway marker. The experiment follows the question: resistance reversal needs causal susceptibility data, mature-biofilm activity needs viable burden in a preformed community, and persistence needs killing and regrowth over time (Brauner et al., 2016; Azeredo et al., 2017; Brauner et al., 2017; Balaban et al., 2019).

### 5.3 Test wound biology before adding formulation complexity

Before adding a carrier, test the combination in mature clinical-isolate *S. aureus*–*P. aeruginosa* or polymicrobial communities under hypoxia, diabetic chemistry, alkaline pH, protein binding and realistic treatment durations. Ex vivo skin lacks perfusion and recruited immunity, while unsplinted mouse wounds close mainly by contraction; splinted diabetic or ischaemic wounds and longer porcine studies provide demanding tests (Dunn et al., 2013; Ganesh et al., 2015; Schaudinn et al., 2017). Where activity is lost identifies the problem a formulation must solve (Johani et al., 2018).

Factorial controls can attribute benefit to the carrier, light or photothermal component, or the co-loaded drug. Unbound wound-fluid and tissue profiles should confirm bacterial exposure without compromising repair; concentrations that inhibit re-epithelialisation have little therapeutic value.

### 5.4 Define and standardise the intervention

Botanical provenance raises the standard for chemical definition: identity, purity, stereochemistry, degradants and potency-linked release for purified compounds; authentication, extraction specifications, fingerprints, marker ranges, contaminants and batch equivalence for standardised products. Historical use does not establish safety at purified high doses, in a new carrier or with nephrotoxic or hepatically metabolised antibiotics. **Figure 2B** retains four co-requisite tests: chemical definition, achievable unbound wound exposure, viable clearance with regrowth testing and compatibility with repair.

Safety cannot be inferred from historical use or from antibacterial activity alone. Purified compounds and new carriers require dose-response cytotoxicity, haemolysis and tissue-compatibility testing, together with chemical identity, purity, batch equivalence, release and degradation data. Repeated topical exposure may cause local irritation or sensitisation, whereas systemic absorption can create hepatic, renal or pharmacokinetic interactions with the companion antibiotic. Photoactivated and redox-active systems also require light-only and repeated-exposure controls because the sensitiser, carrier, heat or reactive species may injure keratinocytes, fibroblasts or immune cells.

### 5.5 Design trials around infection control and durable healing

Proof-of-concept trials should define an infection phenotype—for example, debrided diabetic foot ulcers stratified by pathogen and perfusion—instead of pooling unlike wounds, with adjunct and matched vehicle added to the same guideline-concordant antibiotic and wound care. Clinical resolution and recurrence should anchor efficacy, with surgery or amputation, systemic antibiotic exposure, adverse events and durable closure prespecified (**Figure 2C**); tissue burden, culture, spatial microscopy and biomarkers may explain mechanism but cannot replace patient-important outcomes (Sheehan et al., 2003; Staniszewska et al., 2024). Blinding matters when colour or odour may reveal allocation.

Antibiotic sparing is a separate claim, requiring prospective dose or duration reduction and non-inferiority for infection control, resistance and relapse. Spatially patchy biofilm makes one negative biopsy insufficient evidence of clearance, while unstandardised recovery and rechallenge protocols for wound specimens warrant the term persister-targeting potential, not persister eradication.

## 6 Conclusions

Biofilm-associated antibiotic failure in chronic wounds is a problem of physiology and location as much as resistance. Aggregation, oxygen limitation, slow growth, SCVs and intracellular survival can each blunt killing, although their importance varies from wound to wound and rigorously defined persisters remain difficult to demonstrate in patients.

Among the compounds reviewed here, cinnamaldehyde offers the clearest regulatory mechanism and chenodeoxycholic acid links aminoglycoside uptake to killing across difficult MRSA phenotypes. Sodium houttuyfonate–penicillin G, DHNQ–gentamicin and several carrier-enabled pairs have reached infected-wound models, but with very different controls and attribution. These are credible starting points, not members of an established therapeutic class. Much of the remaining literature rests on early-biofilm assays, indirect energetic or efflux measurements, active formulations or photoactivation.

Depth now matters more than another broad screen. A smaller number of combinations should be tested to failure in mature, wound-relevant communities. Viable killing must exceed both monotherapies at an achievable exposure, persist after withdrawal and leave repair intact. Candidates that clear that bar could enter an add-on trial; none has yet earned a role in chronic-wound care.
