# Supplementary material for: Beyond susceptibility testing: traditional Chinese medicine-associated compounds as candidate antibiotic adjuncts in chronic-wound biofilms
Source: Front Cell Infect Microbiol. 2026 Oct 2;16:1991374. doi: 10.3389/fcimb.2026.1991374 (PMC13673334; doi:10.3389/fcimb.2026.1991374)
Supplement: Supplementary file 1 [file DataSheet1.pdf]

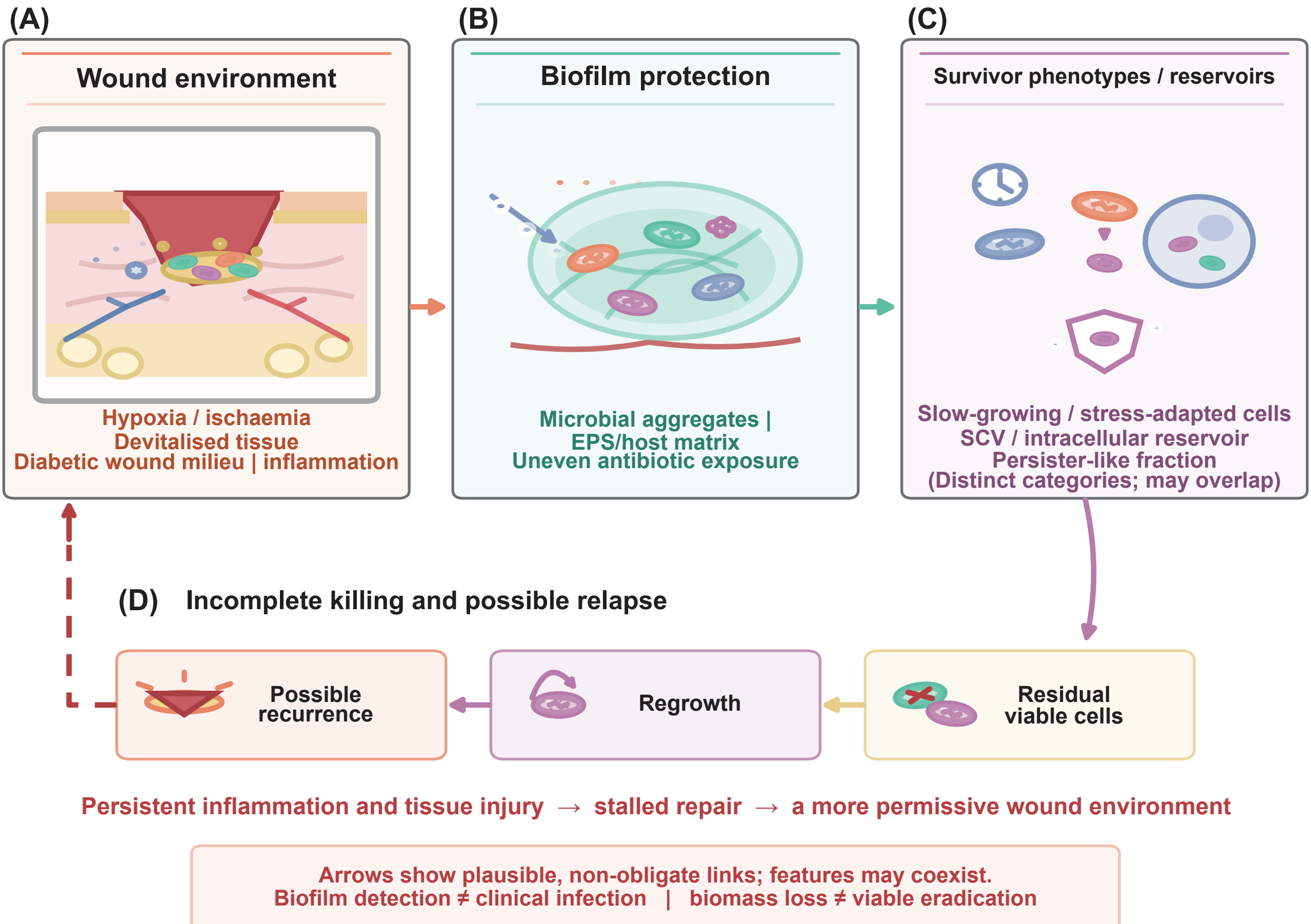

(A)
Wound environment
Hypoxia / ischaemia
Devitalised tissue
Diabetic wound milieu | inflammation
(B)
Biofilm protection
Microbial aggregates |
EPS/host matrix
Uneven antibiotic exposure
(C)
Survivor phenotypes / reservoirs
Slow-growing / stress-adapted cells
SCV / intracellular reservoir
Persister-like fraction
(Distinct categories; may overlap)
(D) Incomplete killing and possible relapse
Possible recurrence
Regrowth
Residual viable cells
Persistent inflammation and tissue injury → stalled repair → a more permissive wound environment
Arrows show plausible, non-obligate links; features may coexist.
Biofilm detection ≠ clinical infection | biomass loss ≠ viable eradication
