# Supplementary material for: Beyond susceptibility testing: traditional Chinese medicine-associated compounds as candidate antibiotic adjuncts in chronic-wound biofilms
Source: Front Cell Infect Microbiol. 2026 Oct 2;16:1991374. doi: 10.3389/fcimb.2026.1991374 (PMC13673334; doi:10.3389/fcimb.2026.1991374)
Supplement: Supplementary file 2 [file DataSheet2.pdf]

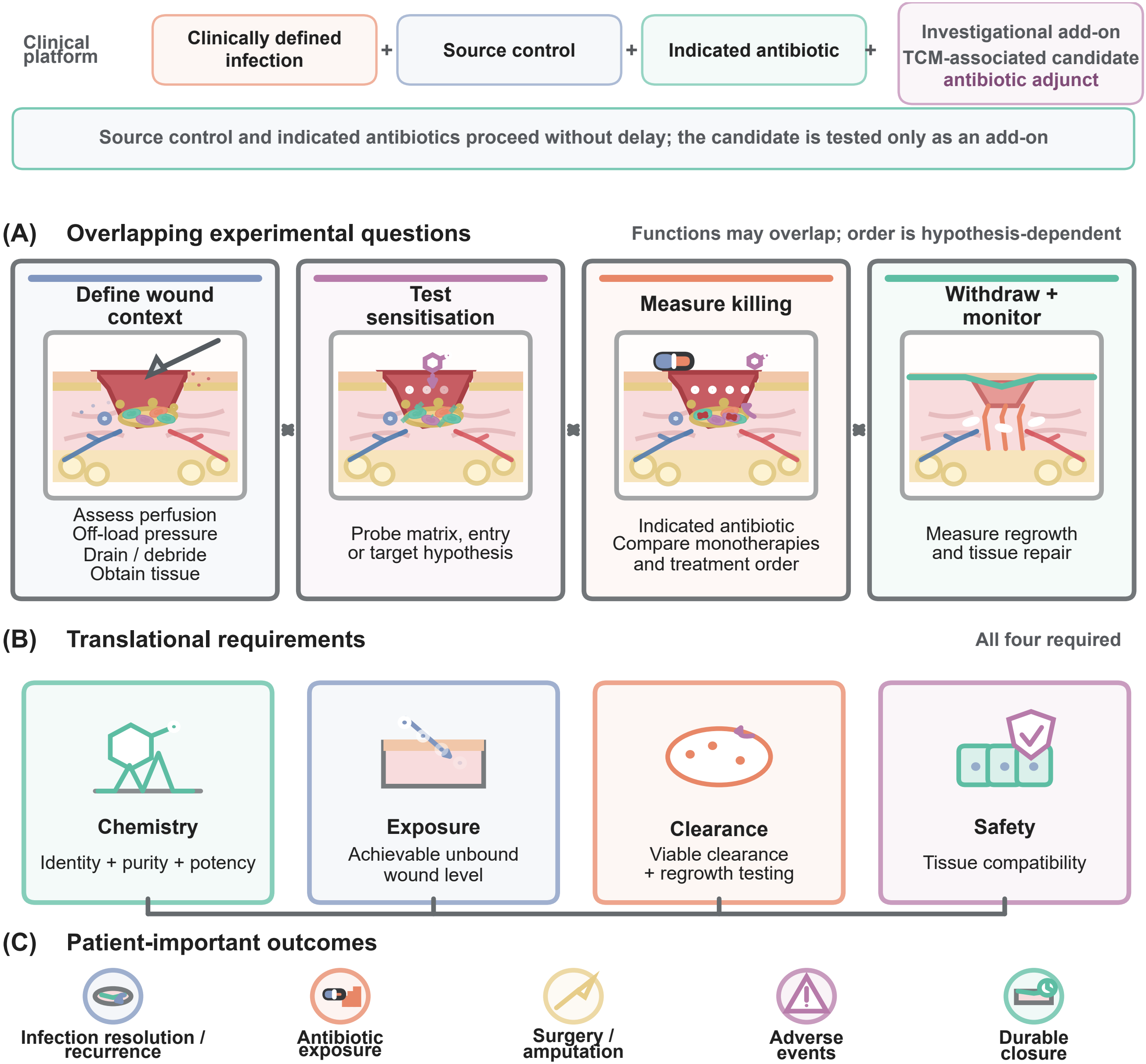
Clinical platform
Clinically defined infection
+
Source control
+
Indicated antibiotic
+
Investigational add-on TCM-associated candidate antibiotic adjunct
Source control and indicated antibiotics proceed without delay; the candidate is tested only as an add-on
(A) Overlapping experimental questions
Functions may overlap; order is hypothesis-dependent
Define wound context
Assess perfusion
Off-load pressure
Drain / debride
Obtain tissue
Test sensitisation
Probe matrix, entry or target hypothesis
Measure killing
Indicated antibiotic
Compare monotherapies and treatment order
Withdraw + monitor
Measure regrowth and tissue repair
(B) Translational requirements
All four required
Chemistry
Identity + purity + potency
Exposure
Achievable unbound wound level
Clearance
Viable clearance + regrowth testing
Safety
Tissue compatibility
(C) Patient-important outcomes
Infection resolution / recurrence
Antibiotic exposure
Surgery / amputation
Adverse events
Durable closure
